# Talarolide A and Talaropeptides A–D: Potential Marine-Derived Therapeutic Peptides with Interesting Chemistry and Biological Activity Studied through Density Functional Theory (DFT) and Conceptual DFT

**DOI:** 10.3390/molecules28186708

**Published:** 2023-09-20

**Authors:** Norma Flores-Holguín, Joan S. Salas-Leiva, Daniel Glossman-Mitnik

**Affiliations:** Departamento de Medio Ambiente y Energía, Centro de Investigación en Materiales Avanzados, Miguel de Cervantes 120, Complejo Industrial Chihuahua, Chihuahua 31136, Mexico; norma.flores@cimav.edu.mx (N.F.-H.); joan.salas@cimav.edu.mx (J.S.S.-L.)

**Keywords:** Talarolide A, Talaropeptides A–D, DFT, Conceptual DFT, KID, cheminformatics, pharmacokinetics

## Abstract

Molecules sourced from marine environments hold immense promise for the development of novel therapeutic drugs, owing to their distinctive chemical compositions and valuable medicinal attributes. Notably, Talarolide A and Talaropeptides A–D have gained recent attention as potential candidates for pharmaceutical applications. This study aims to explore the chemical reactivity of Talarolide A and Talaropeptides A–D through the application of molecular modeling and computational chemistry techniques, specifically employing Conceptual Density Functional Theory (CDFT). By investigating their chemical behaviors, the study seeks to contribute to the understanding of the potential pharmacological uses of these marine-derived compounds. The molecular geometry optimizations and frequency calculations were conducted using the Density Functional Tight Binding (DFTBA) method. This was followed by a subsequent round of geometry optimization, frequency analysis, and computation of electronic properties and chemical reactivity descriptors. We employed the MN12SX/Def2TZVP/H2O model chemistry, utilizing the Gaussian 16 program and the SMD solvation model. The analysis of the global reactivity descriptors arising from CDFT was achieved as well as the graphical comparison of the dual descriptor DD revealing the areas of the molecules with more propensity to suffer a nucleophilic or electrophilic attack. Additionally, Molinspiration and SwissTargetPrediction were considered for the calculation of molecular characteristics and predicted biological targets. These include enzymes, nuclear receptors, kinase inhibitors, GPCR ligands, and ion channel modulators. The graphical results show that Talarolide A and the Talaropeptides A–D are likely to behave as protease inhibitors.

## 1. Introduction

Molecules of marine origin have enormous potential as therapeutic drugs. The oceans are a vast, largely unexplored resource of chemical diversity, and scientists have identified numerous compounds with promising medicinal properties. For example, many marine organisms produce secondary metabolites, such as alkaloids, terpenoids, and polyketides, which have demonstrated anti-cancer, anti-inflammatory, and anti-viral activities. In addition, marine-derived drugs often have unique chemical structures that are not found in terrestrial organisms, making them attractive targets for drug development [1,2]. Despite their potential, only a small fraction of the marine molecules have been thoroughly investigated, due to the challenges of sample collection, extraction, and synthesis. However, advances in technology and research methods, including metagenomics, genomics, and transcriptomics, are making it easier to discover and study new marine compounds. Overall, the potential of molecules of marine origin as therapeutic drugs is vast and largely untapped, offering great promise for the future of medicine [3,4]. Talarolide A and Talaropeptides A–D are a group of natural products that have recently gained attention from the scientific community due to their potential as pharmaceutical drugs. These compounds were first isolated from the marine bacterium *Talaromyces* sp. in 2017, and subsequent research has revealed their interesting chemistry and promising biological activity [5,6,7,8].

Talarolide A is a cyclic depsipeptide that consists of a 16-membered ring with an ester linkage and a lactone ring. It has been found to possess potent anti-inflammatory properties, as well as antitumor activity against several cancer cell lines. Talarolide A is thought to work by inhibiting the NF-κB signaling pathway, which plays a critical role in the regulation of inflammation and cancer [5,6]. Talaropeptides A–D are linear peptides that differ from Talarolide A in their amino acid sequences [7]. Talaropeptide A, for example, has been found to possess antimicrobial activity against several strains of bacteria and fungi. Talaropeptide B, on the other hand, has been found to have weak antitumor activity against cancer cells. Talaropeptides C and D have not been extensively studied yet, but they are expected to have interesting biological properties [7,8].

These compounds have potential as pharmaceutical drugs due to their ability to selectively target specific pathways in the body. For example, Talarolide A may be useful in the treatment of inflammatory diseases such as arthritis, while Talaropeptide A may be useful in the development of new antibiotics to combat drug-resistant bacterial infections. However, further studies are needed to fully understand the pharmacological properties of these compounds and to determine their potential as pharmaceutical drugs [5,6].

In conclusion, Talarolide A and Talaropeptides A–D are a group of natural products that have interesting chemistry and promising biological activity. They have the potential to be developed into pharmaceutical drugs for the treatment of a variety of diseases, but further research is needed to fully realize their potential [9].

Molecular Modeling and Computational Chemistry tools like Conceptual Density Functional Theory (DFT) aid in predicting how chemical reactions occur [10,11,12,13,14,15,16]. This theory uses global and local descriptors to predict the relationship between chemical reactions. The KID (Koopmans in DFT) protocol is used to confirm the model chemistry with the Ionization Energy theorem, and the study found that several descriptors related to HOMO and LUMO calculations are connected to the ΔSCF procedure [17,18,19].

**Figure 1:**
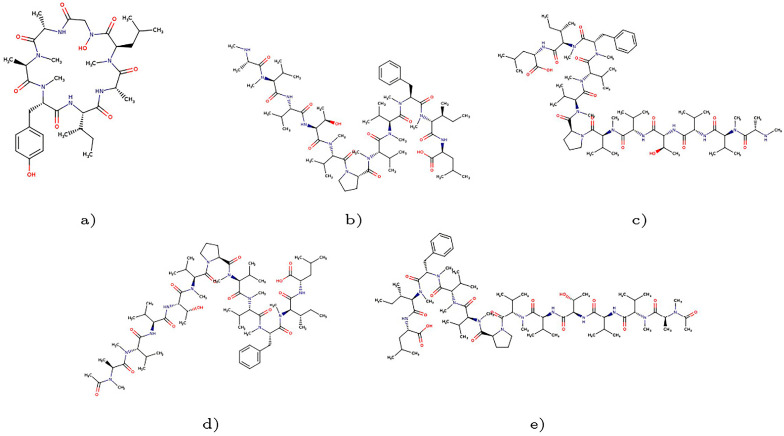
Graphical sketches of the molecular structures of (**a**) Talarolide A, (**b**) Talaropeptide A, (**c**) Talaropeptide B, (**d**) Talaropeptide C and (**e**) Talaropeptide D.

In this study, we focused on these peptides obtained from marine sponges hoping to find a new source of therapeutic peptides. We used Conceptual DFT techniques to predict the chemical reactivity of Talarolide A and Talaropeptides A–D, whose starting molecular structures are shown in Figure 1, involving the determination of local and global properties. This allowed us to predict and understand active reaction sites, both nucleophilic and electrophilic. The study also includes predicting the biological targets using various methods in the literature [9]. The starting molecular structures of the studied peptides were obtained using PubChem (https://pubchem.ncbi.nlm.nih.gov, accessed on 1 March 2023), which serves as a public repository for information related to chemical substances and their biological activities. The methodology used in this study builds upon previously published results on different families of marine-origin therapeutic peptides. By understanding the chemical reactivity of natural products, new medicines can be developed, and molecular modeling and computational chemistry tools like Conceptual DFT aid in predicting how chemical reactions occur, which is crucial for drug discovery [20].

Thus, the objectives of the current research, utilizing DFT and CDFT techniques, encompass the acquisition of valuable insights and fresh knowledge concerning the chemical reactivity of the peptides under investigation. It is our aspiration that these findings will serve as a foundation for the potential design of therapeutic drugs. Furthermore, an additional aim is to forecast the therapeutic targets associated with these marine peptides, potentially paving the way for the development of novel drugs tailored to specific diseases.

## 2. Results and Discussion

**Figure 2:**
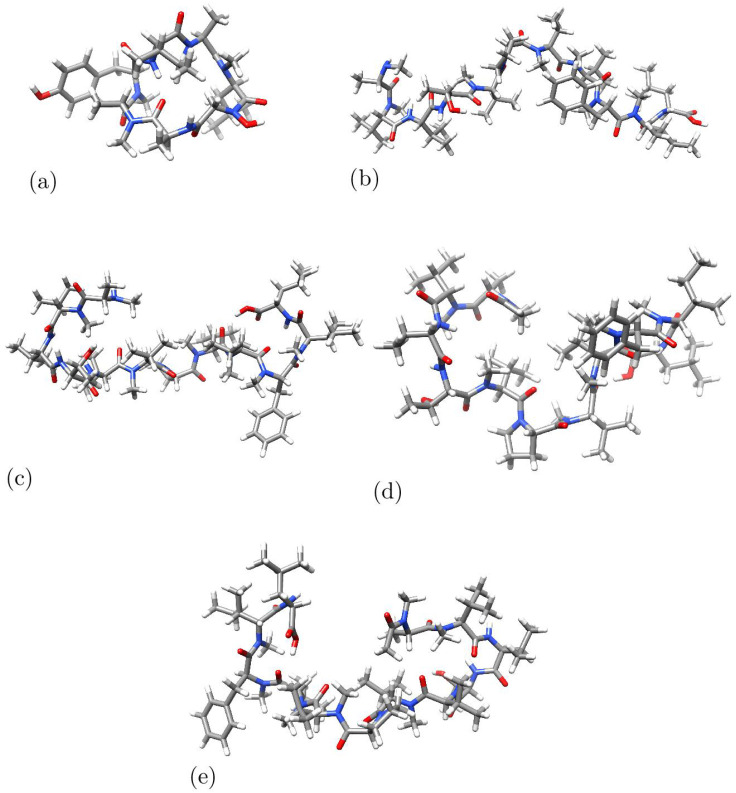
Optimized molecular structures of (**a**) Talarolide A, (**b**) Talaropeptide A, (**c**) Talaropeptide B, (**d**) Talaropeptide C and (**e**) Talaropeptide D.

As outlined in Section 3, we acquired the molecular configurations of the optimized variations of Talarolide A and Talaropeptides A–D sequences. This accomplishment was facilitated by the utilization of the Density Functional Tight-Binding Approximation (DFTBA) model available within Gaussian 16. An initial reoptimization was carried out in the gaseous phase. Subsequently, another reoptimization was conducted through the implementation of the MN12SX density functional, using the Def2SVP basis set and the SMD solvent model with water as the solvent. To ensure congruence with the lowest energy conformations, the frequency-calculation analysis technique was employed for structural validation. However, this time we employed the Def2TZVP basis set instead of the one employed in the initial geometry optimization. The choice of the MN12SX density functional and the Def2SVP basis set for the geometry optimization and the Def2TZVP basis set for the calculation of the Conceptual DFT descriptors is justified on the knowledge acquired through previous published research where it has been shown that these model chemistries are the most adequate on each case for the computational determinations when molecules and peptides of the size of those studied here are considered [17,18,19]. The optimized molecular structures of the studied peptides are displayed in Figure 2.

The aim of the study’s analysis was to verify adherence to the KID (Koopmans in DFT) protocol. During the previous examination, various characteristics linked to the outcomes of HOMO and LUMO calculations were found to correlate with findings obtained through the vertical I and A following the ΔSCF approach. This approach calculates the differences between the energies of the neutral and the ionized species obtained through an SCF calculation. The interrelation between the three main characteristics of the KID procedure and the basic fulfillment of the Janak and Ionization Energy theorems [21,22,23,24,25] is established by linking $\epsilon_{H}$ to -I, $\epsilon_{L}$ to -A, and $J_{I}=\epsilon_{H}+E\left(N-1\right)-E\left(N\right)$, $J_{A}=\epsilon_{L}+E\left(N\right)-E(N+1)$, and $J_{HL}=\sqrt{{J_{I}}^{2}+{J_{A}}^{2}}$. An additional KID descriptor ΔSL defined as the difference between the orbital energies of the HOMO of the radical anion (SOMO) and the LUMO of the neutral system has also been considered for testing how well the model chemistry employed in this work for the determination of the Conceptual DFT reactivity descriptors fulfilled the mentioned theorems [17,18,19]. The findings of this analysis are presented in Table 1:

From Table 1, it can be seen that the values for the KID descriptors are very close to zero. This represents a reasessment that the MN12SX density functional and it derived model chemistry MN12SX/Def2TZVP/H_2_O constitute a wonderful tool for DFT calculations that are in agreement with the Janak and Ionization Energy theorems [21,22,23,24,25]. This is equivalent to say that the MN12SX density functional has a Koopmans-complaint behavior and that the frontier orbital energies determined through its use are enough accurate for the calculation of the chemical reactivity properties that arise from Conceptual DFT.

Considering the KID technique (for Koopmans in DFT) used on the previous studies being integrated into the finite difference approximation [17,18,26], the following expressions can be used to define the global reactivity descriptors [10,12,27,28]:
Electronegativity    $\chi =-\frac{1}{2}\left(I+A\right)\approx \frac{1}{2}\left(\epsilon_{L}+\epsilon_{H}\right)$


Global Hardness    $\eta =\left(I-A\right)\approx \left(\epsilon_{L}-\epsilon_{H}\right)$


Electrophilicity    ω = $\frac{\mu^{2}}{2\eta }=\frac{{\left(I+A\right)}^{2}}{4(I-A)}\approx \frac{{\left(\epsilon_{L}+\epsilon_{H}\right)}^{2}}{4(\epsilon_{L}-\epsilon_{H})}$

Electrodonating Power    $\omega^{-}$ = $\frac{{\left(3I+A\right)}^{2}}{16(I-A)}\approx \frac{{\left(3\epsilon_{H}+\epsilon_{L}\right)}^{2}}{16\eta }$

Electroaccepting Power    $\omega^{+}$ = $\frac{{\left(I+3A\right)}^{2}}{16(I-A)}\approx \frac{{\left(\epsilon_{H}+3\epsilon_{L}\right)}^{2}}{16\eta }$

Net Electrophilicity    $\Delta \omega^{\pm }=\omega^{+}-\left(-\omega^{-}\right)=\omega^{+}+\omega^{-}$



being $\epsilon_{H}$ and $\epsilon_{L}$ the HOMO and LUMO energies associated to each molecule.

Electronegativity, a fundamental concept within Conceptual Density Functional Theory (CDFT), refers to the ability of an atom within a molecule to attract electrons towards itself. In CDFT, electronegativity is not treated as a fixed property but is understood as a response of the electronic system to an external perturbation [10,11,12,13,14,15,16,29].

CDFT goes beyond traditional definitions of electronegativity by considering the change in electron density resulting from an infinitesimal change in electron number, allowing for a more nuanced understanding of how atoms influence each other in a molecule. This approach connects electronegativity to quantities like the chemical potential, which represents the energy change when an electron is added to a system.

By employing CDFT’s framework, researchers can gain insights into reactivity, chemical bonding, and charge transfer in complex systems. This approach enables a deeper exploration of how electronegativity impacts molecular properties and interactions, providing a more refined understanding of chemical behavior at the quantum level.

From Table 2, it can be seen that the electronegativity χ is the same for Talarolide A and Talaropeptides A and B, while is a bit higher for the other two Talaropeptides of the group.

Chemical hardness, a key concept within Conceptual Density Functional Theory (CDFT), refers to the resistance of a molecular system to changes in electron density when subjected to an external perturbation. It is closely related to the concepts of electronegativity and the chemical potential [10,11,12,13,14,15,16,29].

In CDFT, chemical hardness is a measure of the energy required to modify the electron density of a system by a small amount. A molecule with high hardness will require a substantial amount of energy to alter its electron distribution, indicating its stability and unreactive nature. On the other hand, a low-hardness molecule readily adjusts its electron density, making it more prone to interacting with other molecules.

In a similar way to was indicated for the electronegativity χ, we can recall from Table 2 that the chemical hardness η for Talarolide A and Talaropeptide B will be almost of the same value, being Talaropeptide A a system with a little bit higher result. The same can be said about Talaropeptides C and D, being this last one the molecule with the largest η.

Global electrophilicity ω is a fundamental concept within Conceptual Density Functional Theory (CDFT) that characterizes the reactivity of a molecular system towards electron-donating species [30,31,32]. It provides valuable insights into the potential for a molecule to undergo chemical reactions and form new bonds.

In CDFT, global electrophilicity ω is quantified as a function of the chemical potential and the global hardness of a system. It represents the ability of a molecule to accept electrons, making it a measure of its susceptibility to nucleophilic attack or interactions with electron-rich species. Molecules with higher global electrophilicity are more likely to react with nucleophiles, facilitating the formation of new chemical bonds.

Global electrophilicity is an essential tool for assessing and comparing the reactivity of various molecular systems, enabling a deeper understanding of their behavior and interactions within the framework of Conceptual Density Functional Theory.

It is interesting to appreciate from Table 2, that the comparison of the values of ω for the considered peptides follows the same pattern as shown for η, being Talaropeptide C the molecule presenting its large value of this reactivity descriptor.

The global softness S is defined as the reciprocal of η. Thus, the comparison between the different molecules considered in this research is straightforward, rendering values ranging around 0.18–0.19 eV^−1^.

The nucleophilicity index N proposed by Domingo et al. [33,34,35,36,37] is a significant concept within the context of Conceptual Density Functional Theory (DFT), aimed at quantifying and predicting the nucleophilic reactivity of molecules. This index offers valuable insights into the ability of a species to donate electrons and engage in chemical reactions with electrophiles.

The nucleophilicity index N serves as a predictive tool for assessing and comparing the nucleophilic reactivity of different molecular systems. It aids researchers in identifying potential nucleophiles and understanding the driving forces behind nucleophilic reactions. This information is invaluable in various fields, such as organic synthesis, catalysis, and medicinal chemistry, where predicting and controlling reactivity is crucial.

According to the scale proposed by Domingo et al. [33,34,35,36,37] and after an inspection of Table 2, it can be concluded that the five peptides considered in this work may be regarded as moderate nucleophiles.

In the realm of Conceptual Density Functional Theory (DFT), the concepts of electrodonating and electroaccepting powers ($\omega^{-}$ and $\omega^{+}$, respectively) play a crucial role in understanding molecular properties and reactivity. These concepts stem from the idea of using the electron density distribution within a molecule to elucidate its behavior in chemical reactions [27].

Electrodonating power $\omega^{-}$ refers to a molecule’s tendency to donate electrons. It’s characterized by the ability of a molecule to release electron density towards other molecular fragments or atoms, making it more likely to act as a nucleophile in reactions. This is often associated with the presence of lone pairs or π-electron systems that can readily be shared with electron-deficient species [27].

On the other hand, electroaccepting power $\omega^{+}$ signifies a molecule’s capacity to accept electrons. Molecules with high electroaccepting power tend to be electron-deficient or electron-seeking entities. These molecules can attract and stabilize electron density from other molecules or atoms, making them prone to acting as electrophiles in reactions.

For all the molecules considered in this work, it can be seen from Table 2 that their electrodonating powers $\omega^{-}$ are larger than their electroaccepting powers $\omega^{+}$. These result give us a clear idea that how these molecules will behave during a chemical reaction. By comparing the individual peptides, we can conclude that Talaropeptide C displays the largest values of both reactivity descriptors.

The net electrophilicity $\Delta \omega^{\pm }$ is a concept proposed by Chattaraj as a means of comparing the relativer values of $\omega^{-}$ and $\omega^{+}$ [28]. From Table 2, it can be seen that the results for this descriptor will be similar for Talarolide A and Talaropeptide B, and a bit lower for Talaropeptide A. Higher values will be displayed for Talaropeptides C and D, with the former presenting the largest result for these important reactivity descriptor.

The global descriptors demonstrate the chemical reactivity of each molecule in its entirety; therefore, local reactivity descriptors have been designed to assess the differences in the chemical reactivity between the areas inside a molecule. The Dual Descriptor DD [14,38,39,40,41,42] is one of the most important local reactivity descriptors. It has been defined as DD = ${\left(\partial \;f(r)/\partial \;N\right)}_{\upsilon (r)}$, that is, it is related to the Fukui functions, which are other important local reactivity descriptors [10,11,12,13,14,15,16]. Within the realm of Density Functional Theory (DFT), the dual descriptor DD has emerged as an influential instrument harnessed for the exploration of molecular reactivity. This sophisticated metric not only evaluates a molecule’s nucleophilic and electrophilic tendencies but also offers invaluable insights into forecasting chemical reactivity. Its multi-faceted utility spans across various domains of chemistry, encompassing catalyst design, chemical reaction prognostication, and in-depth reactivity analysis. The dual descriptor empowers researchers by furnishing a powerful lens through which to decipher molecular electronic structures, ultimately enabling accurate predictions of their behavior in intricate chemical transformations [38,39,40,41,42].

The computation of the dual descriptor DD stems from the intricate interplay between the energy levels of the Highest Occupied Molecular Orbital (HOMO) and the Lowest Unoccupied Molecular Orbital (LUMO), thereby intricately connected to the concept of the HOMO-LUMO gap. Leveraging the dual descriptor DD equips scientists with the ability to anticipate a molecule’s receptiveness to either nucleophilic or electrophilic attacks. A prominently positive dual descriptor value signifies the molecule’s prowess as an electrophile of note, whereas a significantly negative value denotes its aptitude as a potent nucleophile [38,39,40,41,42].

**Figure 3:**
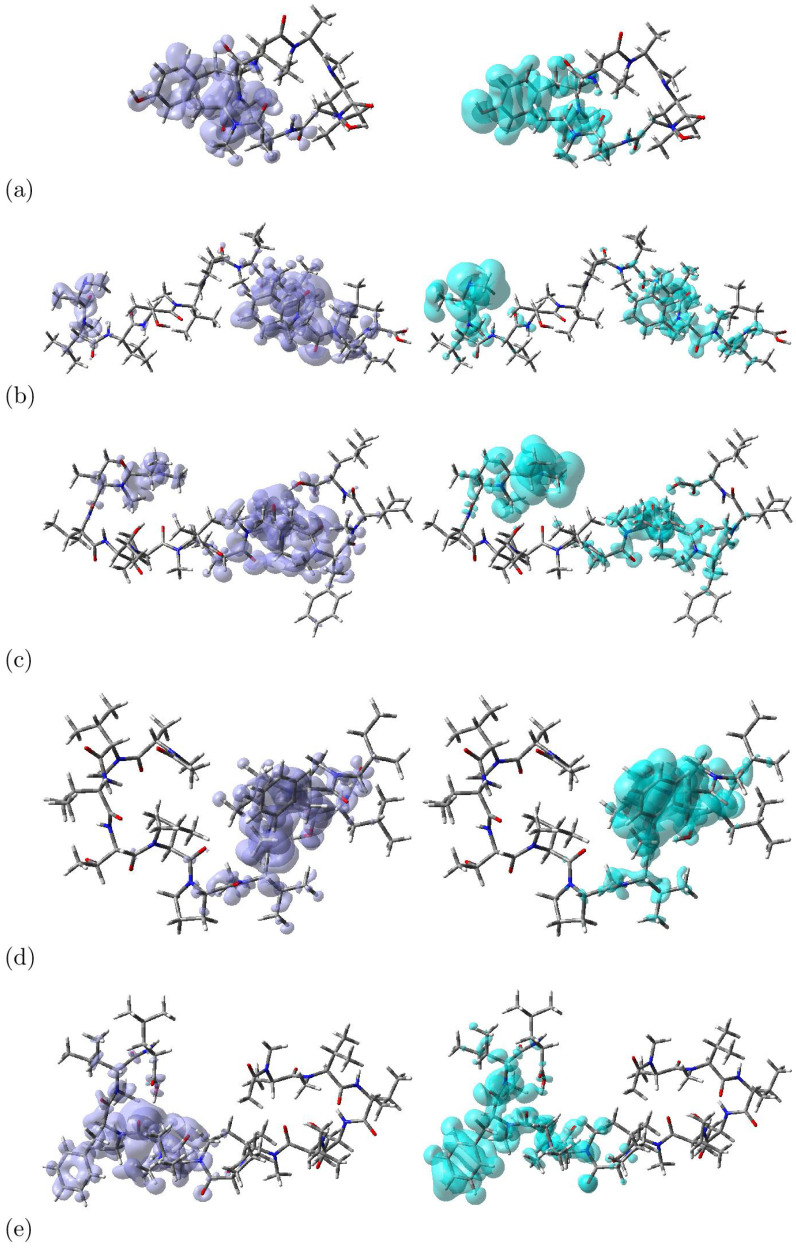
Graphical Representation of the Dual Descriptor DD of (**a**) Talarolide A, (**b**) Talaropeptide A, (**c**) Talaropeptide B, (**d**) Talaropeptide C and (**e**) Talaropeptide D. Left: DD > 0, Right: DD < 0.

**Figure 4:**
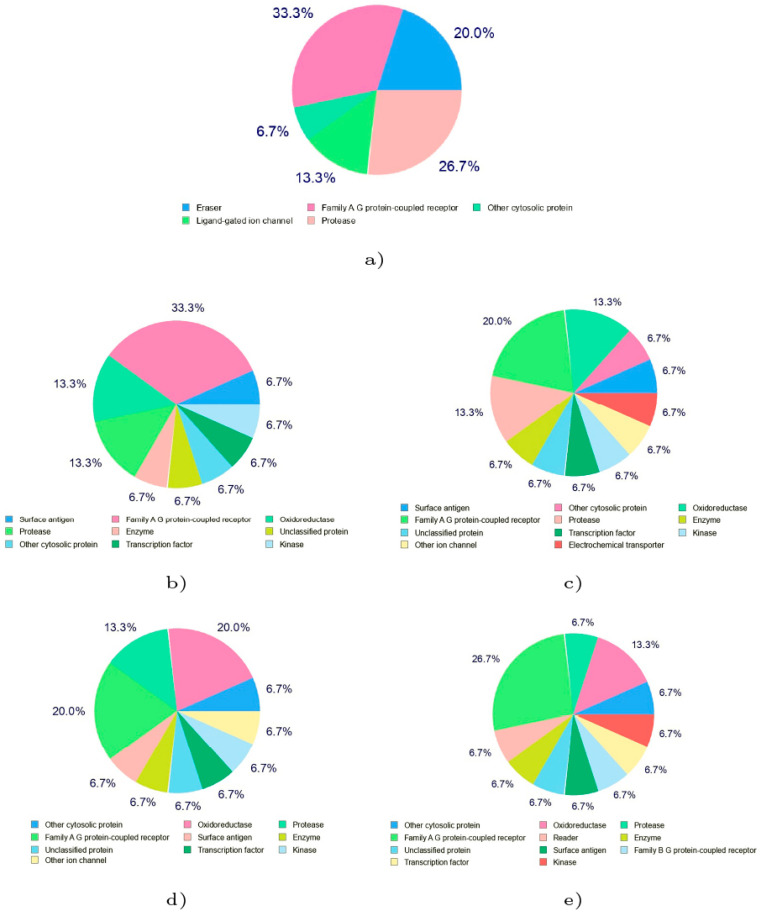
Graphical Representation of the Biological Targets for (**a**) Talarolide A, (**b**) Talaropeptide A, (**c**) Talaropeptide B, (**d**) Talaropeptide C and (**e**) Talaropeptide D.

Graphical representations of the dual descriptor DD for the considered peptides are displayed in Figure 3, where the nucleophilic and electrophilic regions can be distinguished:

As a supplement to the data gathered concerning the chemical reactivity of the investigated marine peptide via CDFT analysis, Figure 4 visually displays the bioactivity rankings of these peptides in relation to different targets. These targets encompass GPCR (G-protein-coupled receptor) ligands, ion channel modulators, nuclear receptor ligands, kinase inhibitors, protease inhibitors, and enzyme inhibitors. The assigned bioactivity scores offer significant perspectives into the possible medicinal uses of these substances [9].

GPCRs are a class of proteins that play a key role in cell signaling and are involved in a wide range of physiological processes. The bioactivity scores presented in Figure 4 suggest that all the considered peptides have potential as GPCR ligands. Their scores as a GPCR ligands indicate that they may be able to selectively bind to and activate specific GPCRs involved in disease processes. This could help in the designing of an effective drug or therapy for treating conditions such as inflammation or cancer [9].

Proteases play crucial roles in numerous biological functions, including protein digestion, blood clotting, and immune responses. Irregular protease behavior has been associated with various medical conditions like viral infections, cancer, and inflammation. When a protease inhibitor binds to an active protease, it impedes its capacity to cleave intended proteins, effectively halting subsequent signal pathways. This action can hinder virus replication, cancer advancement, and inflammatory pathway activation, resulting in beneficial therapeutic outcomes. Targeting protease inhibitors is a key focus in drug exploration and advancement, given their potential to precisely target and restrain specific proteases implicated in disease mechanisms. This leads to more efficient and less hazardous treatment options. Many successful antiviral medications, including HIV protease inhibitors, fall under the category of protease inhibitors [9]. Figure 4 shows that Talarolide A and the Talaropeptides A–D will also display behavior relating to act as proteases inhibitors.

## 3. Materials and Methods

We utilized the Conceptual DFT (CDFT) approach [10,11,12,13,14,15,16,29] to conduct computations for determining molecular energies, electronic densities, and orbital energies of the investigated ligands. We specifically examined the Highest Occupied Molecular Orbital (HOMO) and the Lowest Unoccupied Molecular Orbital (LUMO). Molecular Mechanics (MM) calculations were carried out to identify the conformers of the compounds. For this purpose, we employed the entire MMFF94 force field within MarvinView 17.15 by ChemAxon (http://www.chemaxon.com (accessed on 1 March 2023)) [43,44,45,46,47].

To start, we initiated an optimization of molecular geometry and frequency calculation using the Density Functional Tight Binding (DFTBA) method [48]. This was followed by a subsequent round of geometry optimization, frequency analysis, and computation of electronic properties and chemical reactivity descriptors. We employed the MN12SX/Def2TZVP/H2O model chemistry for these computations [49,50,51], employing the Def2TZVP basis set [50,51]. The molecule’s charge was set to zero, considering the doublet spin state for the radical anion and cation. The MN12SX screened-exchange density functional [49] was integrated into this model. The absence of imaginary frequencies was taken as a criterion to ensure the optimized structure represented an energy minimum. The Gaussian 16 program [48] and the SMD solvation model [52] were used for this purpose.

Understanding the pharmacokinetics, which involves the behavior of a chemical within the body, is crucial for developing new therapeutic drugs [9]. To achieve this, we considered the use of Chemicalize, a tool developed by ChemAxon (http://www.chemaxon.com, accessed on 1 March 2023). Additionally, we employed Molinspiration software (https://www.molinspiration.com/, accessed on 1 March 2023) and SwissTargetPrediction (http://www.swisstargetprediction.ch/, accessed on 1 March 2023) [53] to calculate molecular characteristics and to predict biological targets, including enzymes, nuclear receptors, kinase inhibitors, GPCR ligands, and ion channel modulators.

## 4. Conclusions

In conclusion, this study has highlighted the substantial potential that marine-derived molecules hold for the development of innovative therapeutic drugs. Talarolide A and Talaropeptides A–D have emerged as promising candidates due to their unique chemical compositions and valuable medicinal properties. Through the application of molecular modeling and computational chemistry techniques, particularly Conceptual Density Functional Theory (DFT), the research aimed to delve into the chemical reactivity of these compounds.

By scrutinizing the chemical behaviors of Talarolide A and Talaropeptides A–D, the study sought to enhance our understanding of their possible pharmacological applications. Employing Conceptual DFT and other computational tools, the investigation revealed the chemical reactivity properties, active reaction sites through the dual descritor DD and potential interactions with diverse biological targets.

In summary, this research underscores the importance of comprehending the chemical reactivity of natural marine products in advancing pharmaceutical development. The insights gleaned from this study, facilitated by computational methodologies like Conceptual DFT, shed light on active sites and potential biological targets of Talarolide A and Talaropeptides A–D. These findings contribute to the broader understanding of marine-derived peptides and their promising roles as pharmaceutical agents. Nevertheless, further investigations are imperative to fully unravel the complete range of their pharmacological attributes.

## Figures and Tables

**Table 1:** Orbital energies, H-L gap and the KID descriptors (all in eV) for the Talarolide A and Talaropeptides A–D molecules.

	HOMO	LUMO	SOMO	H-L Gap	J(I)	J(A)	J(HL)	ΔSL
Talarolide A	−6.14	−0.93	−0.96	5.21	0.04	0.02	0.05	0.02
Talaropeptide A	−6.22	−0.85	−0.87	5.37	0.02	0.00	0.02	0.01
Talaropeptide B	−6.16	−0.92	−0.92	5.24	0.00	0.00	0.00	0.00
Talaropeptide C	−6.54	−1.00	−1.03	5.54	0.01	0.01	0.02	0.02
Talaropeptide D	−6.56	−0.92	−0.91	5.63	0.01	0.00	0.01	0.01

**Table 2:** Global reactivity descriptors for the Talarolide A and Talaropeptide A–D molecules (all in eV, excepting S, in eV^−1^).

	χ	η	ω	S	N	$\omega^{-}$	$\omega^{+}$	$\Delta \omega^{\pm }$
Talarolide A	3.54	5.21	1.20	0.19	2.65	4.50	0.96	5.46
Talaropeptide A	3.54	5.37	1.17	0.19	2.57	4.44	0.90	5.34
Talaropeptide B	3.54	5.24	1.20	0.19	2.63	4.50	0.95	5.45
Talaropeptide C	3.77	5.54	1.29	0.18	2.25	4.80	1.03	5.83
Talaropeptide D	3.74	5.63	1.24	0.18	2.24	4.70	0.96	5.67

## Data Availability

All generated data will be available from the authors under request.
